# The Relationship Between Obstructive Sleep Apnea (OSA) and Gastroesophageal Reflux Disease (GERD) in Inpatient Settings: A Nationwide Study

**DOI:** 10.7759/cureus.22810

**Published:** 2022-03-03

**Authors:** Ratib Mahfouz, Andriy Barchuk, Adham E Obeidat, Mahmoud M Mansour, David Hernandez, Mohammad Darweesh, Mohammad Aldiabat, Mohannad H Al-Khateeb, Mubarak H Yusuf, Yazan Aljabiri

**Affiliations:** 1 Internal Medicine, Kent Hospital/Brown University, Warwick, USA; 2 Internal Medicine, Kent Hospital, Warwick, USA; 3 Internal Medicine, University of Hawaiʻi, Honolulu, USA; 4 Internal Medicine, University of Missouri School of Medicine, Columbia, USA; 5 Internal Medicine, Brown University, Providence, USA; 6 Internal Medicine, East Tennessee State University, Johnson City, USA; 7 Internal Medicine, New York City Health and Hospitals Corporation (NYCHHC) Lincoln Medical Center, New York, USA

**Keywords:** obstructive sleep apnea, gerd pathophysiology, prevalance, prevalence of gerd, osa

## Abstract

Introduction: Several studies identified a link between gastroesophageal reflux disease (GERD) and obstructive sleep apnea (OSA). GERD is a condition in which acid reflux from the stomach to the esophagus causes troublesome symptoms. On the other hand, OSA is defined as a sleep-related breathing disorder in which airflow significantly decreases or ceases due to upper airway obstruction, leading to arousal from sleep. OSA was found to be associated with GERD. In this study, we aim to study the characteristics and concurrent risk factors associated with GERD and OSA in a large population-based study.

Methods: Patients with the diagnosis of GERD were extracted from the National Inpatient Database (NIS) for the years 2016 to 2019. Patients' age, gender, race, and hospital information, including region and bed size, were extracted and considered as baseline characteristics. The comorbidities included are hypertension (HTN), atrial fibrillation (AFib), congestive heart failure (CHF), chronic obstructive pulmonary disease (COPD), pulmonary hypertension (PHTN), obesity, and smoking. Patients younger than 18 years old were excluded from this study.

Results: Out of 22,677,620 patients with the diagnosis of GERD, 12.21% had a concurrent diagnosis of OSA (compared to 4.79% in patients without GERD, p-value <0.001). The mean age of patients with GERD and OSA was 64.47 years vs 65.42 years in patients without OSA (p-value <0.001). The GERD and OSA group had almost identical gender distribution compared to the GERD only group, as it was predominantly female patients. The white and black races were slightly more prevalent in the GERD and OSA group compared to the GERD only group. Regarding comorbidities, the prevalence of obesity was more clear in the GERD and OSA group. It was noted that the group of patients who carry a diagnosis of GERD and OSA have more prevalence of diabetes (DM), hypertension (HTN), obesity, atrial fibrillation (Afib), congestive heart failure (CHF), and pulmonary hypertension (PHTN). Patients with GERD and OSA were 21% less likely to be older than 65 years rather than younger (95% CI: 0.79-0.8, p-value <0.001), 35% less likely to be females (95% CI: 0.65-0.65, p-value <0.001), and 22% less likely to be non-white (95% CI: 0.77-0.8, p-value <0.001). Obesity was found to be the strongest association with this population, followed by PHTN, CHF, DM, HTN, Afib, and lastly smoking.

Conclusion: Patients with GERD and OSA were found more likely to be female, white, living in the southern part of the United States, obese, diabetes mellitus type 2, and being active smokers.

## Introduction

Several studies identified a link between gastroesophageal reflux disease (GERD) and obstructive sleep apnea (OSA) [1,2]. GERD is a condition in which acid reflux from the stomach to the esophagus causes troublesome symptoms (typically including heartburn or regurgitation) and/or esophageal complications. Globally, GERD affects 8% to 33% of people, affecting both genders and all age groups [2]. It is mainly diagnosed clinically based on reported symptoms, but sometimes specific examinations such as impedance-pH monitoring and gastroscopy can support the diagnosis [3]. Known complications of GERD include erosive esophagitis, Barrett's esophagus, esophageal stricture, and asthma [4,5]. On the other hand, OSA is defined as a sleep-related breathing disorder in which airflow significantly decreases or ceases due to upper airway obstruction, mainly in the oropharynx, leading to arousal from sleep. As a result, partial pressure of oxygen in arterial blood (PaO_2_) is decreased, while partial pressure of carbon dioxide (PaCO_2_) is increased. This leads to complications such as hypoxic pulmonary vasoconstriction leading to pulmonary hypertension and cor pulmonale along with secondary hypertension in the setting of increased sympathetic activity [6]. 

According to the Wisconsin cohort study, the prevalence of OSA in people aged 30 to 60 years ranges from 9% to 24% in men and 4% to 9% in women [7]. Usually, the main complaints are chronic fatigue, nocturia, poor concentration, and depressed mood [8]. As the reported symptoms and physical exam can be nonspecific or not indicative, several tools were developed to identify patients with a potential diagnosis of OSA, such as Epworth Sleepiness Scale (ESS) and Stop-Bang Questionnaires. The diagnosis is mainly confirmed by polysomnography (PSG) [9]. Cardiovascular diseases, stroke, and diabetes risk were found to be increased with OSA [10]. OSA and GERD have a strong relationship. Approximately 40%-60% of people with OSA also suffer from GERD, which can be extremely difficult to treat and result in extremely disruptive sleep patterns [11,12]. In this study, we aim to study the characteristics and concurrent risk factors associated with GERD and OSA in a large population-based study.

## Materials and methods

Data source

This is a retrospective cohort study of patients who were admitted to hospitals in the United States between the years 2016 and 2019. The data were extracted from the Healthcare Cost and Utilization Project National Inpatient Sample (NIS) database. The NIS is sponsored by the Agency for Healthcare Research Quality (AHRQ) and is considered the largest publicly available inpatient healthcare database in the United States. The database includes data from at least 46 states and covers more than 97% of the US population [13]. A 20% probability sample was collected and subsequently weighted to ensure that the selected population was nationally representative. Each admission in the database was assigned one principal diagnosis, up to 40 secondary diagnoses, and 25 procedures. These variables are defined via the International Classification of Disease, 10th Revision, and Clinical Modification (ICD-10-CM) codes. 

Study variables

**Figure 1:**
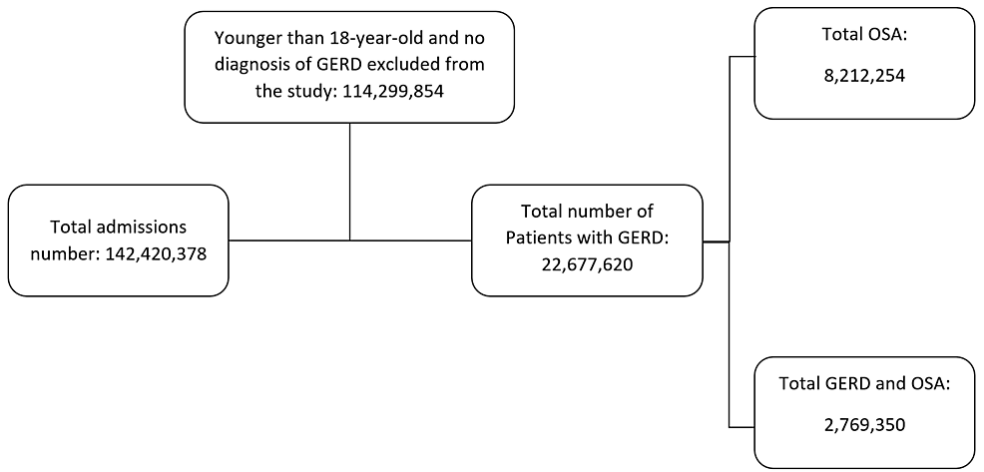
Flow diagram of patients selection. GERD, gastroesophageal reflux disease; OSA, obstructive sleep apnea.

Using ICD-10-CM codes, we were able to identify patients who carry diagnoses of OSA and GERD. Patients' age (in years), gender, race (white, black, Hispanic, others), and hospital information (region and bed size) were collected and considered as baseline characteristics. The comorbidities included are hypertension (HTN), atrial fibrillation (AFib), congestive heart failure (CHF), chronic obstructive pulmonary disease (COPD), pulmonary hypertension (PHTN), obesity, and smoking. Patients younger than 18 years old were excluded from this study. Patients with a body mass index (BMI) of more than 24.9 were considered obese. A flow chart of the study population selection process is demonstrated in Figure 1.

Statistical analysis

The statistical analysis was done using STATA software, version 17.0 (StataCorp., College Station, TX, USA). The characteristics of patients with OSA alone and those who had both OSA and GERD were described using descriptive statistics. In this study, multivariate logistic regression analyses were performed to determine factors associated with in-hospital mortality. Variables that were not statistically significant (p-value <0.1) on univariate analysis were excluded from the multivariate analysis. The odds ratio at 95% CI was used to describe the association between the study and outcome variables. Statistical significance was defined as a two-tailed p-value of <0.05.

## Results

Demographic and clinical characteristics

Out of 22,677,620 patients carrying the diagnosis of GERD, it was found that 12.21% were diagnosed with concurrent OSA (compared to 4.79% in patients without GERD, p-value <0.001) (Figure 1). The mean age of patients with GERD and concurrent OSA was slightly less than that of patients with GERD only (64.47 years vs 65.42 years, p-value <0.001). The GERD and OSA group had almost identical gender distribution compared to the GERD only group, as it was predominantly female patients. The white and black races were slightly more prevalent in the GERD and OSA group compared to the GERD only group. In terms of hospital information, the GERD and OSA group was more likely to be in a large hospital in the midwest region compared to the GERD only group, but the southern region remained the most prevalent region in both groups (Table 1). Regarding comorbidities, the prevalence of obesity was more clear in the GERD and OSA group. It was noted that the group of patients who carry a diagnosis of GERD and OSA have more prevalence of DM, HTN, obesity, Afib, CHF, and PHTN.

**Table 1:** Breakdown of characteristics and their statistical significance. GERD, gastroesophageal reflux disease; OSA, obstructive sleep apnea; DM, diabetes; HTN, hypertension; Afib, atrial fibrillation; CHF, congestive heart failure; PHTN, pulmonary hypertension.

Characteristics	GERD only (22,677,620)	OSA and GERD (2,769,350)	P-value
Age (years)	65.42	64.47	<0.0001
Gender (%)			<0.0001
Males	40.38	49.37	
Females	59.62	50.63	
Race (%)			<0.0001
White	75.91	78.27	
Black	12.5	13.65	
Hispanic	7.21	5.3	
Others	4.38	2.78	
Hospital region (%)			
Northeast	19.18	16.82	<0.0001
Midwest	24.06	31.17	
South	41.45	37.09	
West	15.32	14.91	
Hospital bed size (%)			<0.0001
Small	20.93	19.85	
Medium	28.91	27.66	
Large	50.16	52.48	
Comorbidities (%)			<0.0001
Obesity	16.59	45.25	
Smoke	41.8	43.87	
DM	30.37	47.62	
HTN	31	22.42	
Afib	16.61	23	
CHF	19.85	32.33	
PHTN	0.04	0.1	

Potential predictors of concurrent OSA diagnosis in GERD patients 

**Figure 2:**
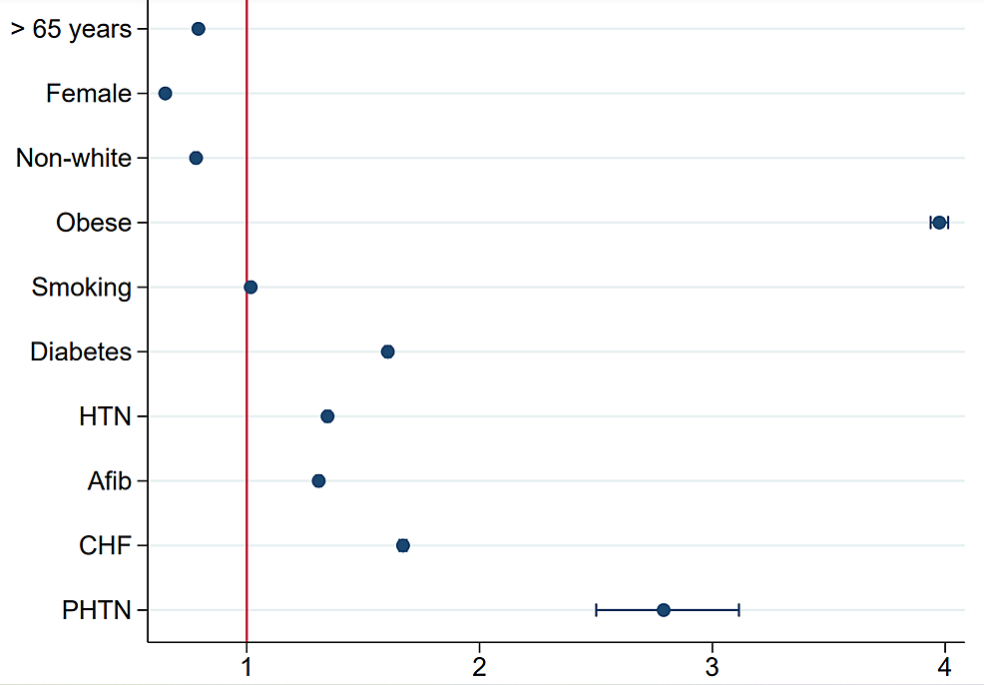
Plot demonstrating predictors of OSA in the GERD population. GERD, gastroesophageal reflux disease; OSA, obstructive sleep apnea; HTN, hypertension; Afib, atrial fibrillation; CHF, congestive heart failure; PHTN, pulmonary hypertension.

These patients were 21% less likely to be older than 65 years rather than younger (95% CI: 0.79-0.8, p-value <0.001), 35% less likely to be females (95% CI: 0.65-0.65, p-value <0.001), and 22% less likely to be non-white (95% CI: 0.77-0.8, p-value <0.001). Obesity was found to be the strongest association with this population, followed by PHTN, CHF, DM, HTN, Afib, and lastly smoking (Table 2). A plot demonstrating predictors of OSA in the GERD population is shown in Figure 2.

**Table 2:** Potential predictors of concurrent OSA diagnosis in GERD patients. OSA, obstructive sleep apnea; GERD, gastroesophageal reflux disease; DM, diabetes; HTN, hypertension; Afib, atrial fibrillation; CHF, congestive heart failure; PHTN, pulmonary hypertension.

Variable	Adjusted OR (95% CI)	P-value
Age		<0.001
18-65 years	Reference	
>65 years	0.79 (0.79-0.8)	
Gender		<0.001
Male	Reference	
Female	0.65 (0.65-0.65)	
Race		<0.001
White	Reference	
Non-white	0.78 (0.77-0.8)	
Comorbidities		<0.001
Obesity	3.98 (3.94-4.01)	
Smoking	1.02 (1.01 -1.03)	
DM	1.6 (1.59-1.62)	
HTN	1.35 (1.34-1.36)	
Afib	1.31 (1.3-1.32)	
CHF	1.67 (1.66-1.69)	
PHTN	2.79 (2.5-3.11)	

## Discussion

This study attempts to further elucidate the relationship between GERD and OSA. Prior studies over the past decade have demonstrated the growing evidence of GERD complications and the importance of employing a multidisciplinary approach to target the most recognized cardiovascular, pulmonary, laryngeal, and dental complications associated with this common medical condition [14]. Only more recently, a potential relationship between GERD and OSA has been studied [15]. One proposed mechanism is that the greater respiratory effort and the cough associated with respiratory conditions increase the pressure gradient across the lower esophageal sphincter (LES), which in turn increases the pressure and promotes the opening of the LES, concomitantly increasing abdominal pressure and ultimately increasing the risk of gastric reflux by facilitating the retrograde movement of gastric contents [16]. Other explanations include hyperinflation interfering with the normal diaphragmatic contribution to the LES, the relaxing effect of bronchodilator medication on the LES function, and the high prevalence of hiatal hernia in these patients [17-19]. Finally, both OSA and GERD share many of the same risk factors. While the prevalence of GERD and OSA is high in the general population, there is currently no explanation of any potential causal relationship between these two conditions. 

In our study, the prevalence of OSA in patients with GERD is more than that in patients without GERD. Moreover, some factors were associated with a higher chance of having OSA in patients with GERD such as female gender, the white race, living in the southern part of the United States, obesity, DM type 2, and active smoking. Obesity correlates well with the risk of OSA [20]. A number of mechanisms may explain the association of excess body mass with OSA, including increased upper airway collapsibility and impaired neuromuscular control [21]. In one study, it was suggested that an increase in weight by 10% could lead to a six-fold increase in OSA risk [22]. Obesity has also been found to be associated with transient relaxations of the lower esophageal sphincter (TRLES). TRLES is primarily triggered by gastric distension, especially in the fundus, and is considered to be one of the main mechanisms for GERD development [23].

Smoking was associated with an increase in nasal airflow resistance, which is hypothesized to be a contributing factor for OSA development [24,25]. Compared to those who had never smoked or who had smoked in the past, current smokers were three times as likely to have OSA [26]. On the other hand, smoking tobacco was found to reduce the LES resting pressure, which can eventually lead to GERD [27]. It is possible that OSA and DM type 2 may share a bidirectional relationship. This is because diabetic neuropathy can alter central control of respiration and upper airway nerves, resulting in OSA [28]. There are several mechanisms contributing to GERD in DM, including motor and LES abnormalities, hormonal changes, extra-abdominal pressure, obesity, and decreased parotid gland secretion, which reduces the clearance of esophageal acid [29].

Strengths and limitations

Several advantages of our study include the use of NIS, the largest publicly available inpatient database, which makes the results more representative since the sample size is large; it adjusts all outcomes to the most common baseline characteristics of both patients and hospitals to minimize confounding factors as much as possible, and it analyzes multiple demographics for patients with GERD and OSA. In our study, we encountered some limitations. First, it is a retrospective study, which makes it susceptible to nonrandomization. Second, the NIS database includes an administrative database, which means that administrative codes were used to identify GERD, OSA, and other diagnoses, leading to possible misclassifications, undercodings, or overcodings. The misclassification will, however, be seen as an error rather than a bias since it is likely to occur equally across all arms. Errors do not change the nature of the relationship between two variables; rather, statistical significance between them becomes more difficult to establish.

## Conclusions

Our study demonstrated a close relationship between GERD and OSA and the characteristics and risk factors that would predispose someone with the diagnosis of GERD to also have OSA. Patients with GERD who carry the highest risk factors being female, white, living in the southern part of the United States, obese, diabetes mellitus type 2, and being an active smoker would likely benefit most from being screened to have a concomitant diagnosis of OSA. As GERD tends to be more symptomatic and more likely to be reported by patients compared to OSA, and given previous literature on the relationship between OSA and GERD that was reinforced by our paper, we propose that OSA screening might be warranted in patients with an underlying diagnosis of GERD, as it is associated with long-term significant morbidity and mortality. Nonetheless, more studies are needed on that content before any definitive conclusion can be made.
